# Avicenna’s Point about Bladder Gas as a Cause of Interstitial Cystitis

**Published:** 2018-09

**Authors:** Malihe TABARRAI, Zahra NIKTABE, Nematollah MASOUDI, Tahereh EFTEKHAAR

**Affiliations:** 1. School of Traditional Medicine, Tehran University of Medical Sciences, Tehran, Iran; 2. School of Traditional Medicine, Shahid Beheshti University of Medical Sciences, Tehran, Iran; 3. Dept. of Pelvic Floor, Imam Khomeini Hospital Complex, Tehran University of Medical Sciences, Tehran, Iran

## Dear Editor-in-Chief

Interstitial cystitis (IC) or bladder pain syndrome (BPS) is considered a devastating condition of chronic nature (1) which can have negative impact on the patients’ quality of life (2). American Urological Association describes the term IC/BPS as “An unpleasant sensation (pain, pressure, discomfort) perceived to be related to the urinary bladder, associated with lower urinary tract symptoms of longer than 6 weeks’ duration, in the absence of infection or other identifiable causes” (3).

The level of distress can be variable from abdominal tenderness to severe bladder spasm (1). The diagnosis of this condition is still not very clear and depends on ruling out other diseases (3). The etiology of BPS is still undetermined mainly due to disagreement on its classification (1). Recent studies show the significant association of environmental factors such as diet, drinking behavior, physical activity and smoking with occurrence of BPS/IC (2).

In this article, we present an interesting and important etiology, which may justify some types of pain in interstitial cystitis.

In historical medical manuscripts, Avicenna (980–1037 AD), the famous Iranian physician, described gas in the bladder as a possible cause of bladder pain and urinary tract symptoms (inability to Urinate normally from the bladder), without infection and other urinary disorders. In this disorder excess moisture (Rotubat) and coldness in temperament of bladder, tissues weaken the bladder cells function which through incomplete metabolism leads to the production of gas trapped in these tissues (4). According to this opinion, entrapped gas can cause pain by stretching and compression of tissues. The site of pain could change according to the displacement of gas bubbles (5, 6). In addition, excessive consumption of flatulent foods is another reason for development of this disease. Avicenna called this disease “the bladder gas” (5). In Iranian traditional medicine, this “Gas” is one of the causes of pain and dysfunction in other organs such as the neck, waist, and kidneys as well (6, 7).

Iranian medicine treatment of this condition generally consists of three major steps: 1. Lifestyle modification 2. Using topical and oral medications 3. Manipulation (aamale yadavi) if needed (5, 8). In the case of bladder Gas Traditional medicine texts such *Al-Qanun fi Tibb* have suggested a wide range of recommendation that includes regimental therapy along with numerous oral and topical medications such as figs and fragrant and solver oils like iris oil and saffron oil (5). Using warm compress and dry cupping is also recommended as well as conventional medicine (1, 4, 5).

In conventional medicine first report of gas in the spine was in 1937 and this view has since been confirmed in a number of studies (6). Moreover, there is report of Intra-osseous gas in CT examinations of patients with sacral insufficiency fracture (9, 10). Pain, spasm, lower urinary tract symptoms, therapeutic response to dietary changes and Warm-up bladder, the absence of infection and other urinary disorders are obvious similarities between Interstitial cystitis in conventional medicine and bladder Gas in traditional medicine (1, 2, 3, 5). A significant overlap exists between the two diseases and clinical study of traditional medicine ideas could open a new window in the treatment of patients with interstitial cystitis.

There has been no previous article with direct suggestion of possibility of the gas as a cause of interstitial cystitis.
